# Rationale, Design, and Baseline Characteristics of the TARGET (Targeted Assessment and Recruitment of Geriatrics for Effective Fall Prevention Treatments) Cohort of Older Adults for Assessing Fall and Fracture Risk: Prospective Cohort Study

**DOI:** 10.2196/89285

**Published:** 2026-09-25

**Authors:** Angelique Chan, Abhijit Visaria, Rahul Malhotra, Navrag B Singh, Benedikt Helgason, Victor R Schinazi, Kok Yang Tan, David B Matchar, Rita Sim, Vanessa Jean Wen Koh, Wei Xuan Lai, Lakkhina Troeung, Catherine Wen Huey Lim, Sai G S Pai, Kai Zhe Tan, Anitha D Praveen, Dheeraj Jha, Stephen J Ferguson, Giorgio Colombo, Karolina Minta, Ecosse L Lamoureux, William R Taylor

**Affiliations:** 1 Centre for Ageing Research & Education (CARE), Duke-NUS Medical School, 8 College Road, Singapore, 169857, Singapore, +6565165685; 2 Health Services Research & Population Health (HSRPH), Duke-NUS Medical School, Singapore, Singapore; 3 Future Health Technologies (FHT), Singapore-ETH Centre, Singapore, Singapore; 4 Institute for Biomechanics, ETH Zurich, Zurich, Zurich, Switzerland; 5 Faculty of Society & Design, Bond University, Gold Coast, Queensland, Australia

**Keywords:** aging, falls, fractures, health technology, predictive algorithms, population health

## Abstract

**Background:**

Falls and fractures are a major clinical concern for older adults. International clinical guidelines recommend annual fall risk screening for all adults aged 60 years and older. However, existing falls risk–screening algorithms have shown limited sensitivity and specificity in detecting future falls. Emerging health technologies, including wearable sensors, in-silico finite element models (FEMs), and virtual reality (VR) technology, show promise in enhancing fall and fracture risk assessments by providing more personalized objective data to support targeted primary prevention. However, large-scale longitudinal studies are required to evaluate their predictive accuracy and cost-effectiveness for systematic community–based screening.

**Objective:**

The TARGET (Targeted Assessment and Recruitment of Geriatrics for Effective Fall Prevention Treatments) study is an ongoing national prospective cohort study in Singapore designed to develop cost-effective and scalable frameworks for the early detection and prevention of falls and fractures using novel health technologies. This paper describes the rationale, design, and baseline characteristics of TARGET, and provides preliminary insights into how these novel technologies perform in discriminating between older adults with and with no history of falls at study baseline.

**Methods:**

A total of 2291 community-dwelling Singapore residents aged ≥60 years were enrolled between 2022 and 2024. Participants underwent a comprehensive baseline fall risk assessment comprising (1) home-based interview to assess sociodemographic, anthropometric, cognitive, physical, functional, and psychosocial status; (2) gait assessment using wearable inertial measurement units (IMUs) motion sensors; (3) dual-energy X-ray absorptiometry and whole-body 3D scans to construct subject-specific FEMs; and (4) VR-based cognitive assessment to probe spatial navigation deficits. Prospective follow-up for 2 years is ongoing, with linkage to national electronic medical records (EMRs) to obtain detailed clinical, medication, and pathological data.

**Results:**

Of the total cohort, 58% (1327/2291) were female, with a mean age of 74.6 years at baseline. TARGET represents a relatively healthy older adult population, with 83% (1898/2291) remaining fully independent, 44% (1016/2290) with mild cognitive impairment, 50% (440/879) with osteopenia, and 15% (358/2291) reporting falls in the past year. Significant baseline functional, psychosocial, cognitive, and biomechanical differences were identified between fallers and nonfallers. Fallers had poorer physical and psychosocial health, greater gait variability, and a higher risk of osteoporotic fractures. Novel risk prediction models integrating epidemiological, IMUs, FEM, VR, and EMR data are in development and being validated against gold standard clinical assessments, with cost-effectiveness evaluations of the economic feasibility of adopting these screening measures at scale.

**Conclusions:**

Leveraging comprehensive epidemiological, EMR, and health technology data, TARGET is well positioned to identify novel biomarkers and systemic interactions that may predict falls and fracture risk, with potential application to other age-related diseases with shared pathophysiology. In particular, TARGET’s focus on community-based screening using technologies that can be administered by nonclinical personnel has the potential to reduce substantial burden and costs within the health care sector.

## Introduction

Falls and fall-related injuries are a major public health and clinical challenge worldwide, especially given the ubiquity of population aging [1]. Approximately 30% of community-dwelling adults aged 65 years or older sustain at least 1 fall each year, increasing to 50% in persons aged older than 80 years [2,3]. Falls are a prominent clinical feature in later-life diseases affecting older adults, defined as persons aged 60 years or older. Notably, falls occur frequently in neurological disorders, affecting up to 70% of individuals with Parkinson disease [4], at least 50% of stroke survivors [5], and 20%‐30% of older adults with peripheral neuropathy [6]. The incidence of falls has been estimated to be 50% among older adults with diabetes, particularly when peripheral neuropathy or hypoglycemia is present [7]. Moreover, falls occur in 30%‐50% of older adults with cardiovascular diseases [8] or cancer, especially among those receiving chemotherapy [9]. Together, these data highlight the considerable significance of falls in the context of aging and disease.

Critically, falls are often a sentinel event for older adults and mark a turning point in the trajectory toward the end of life [10]. A single fall can result in hospital admission, major surgical intervention, and a prolonged convalescence [11]. Recurrent falls are a widely accepted clinical indicator of advanced underlying pathology [12]. Notably, severe acute fall-related injuries such as hip fractures often trigger cascading complications, including prolonged hospitalization and secondary infections [13-15]. The 12-month mortality rate following a hip fracture is between 22% and 29% with excess mortality observed up to 10 years postinjury compared with age-matched controls [16]. In the United States and Europe alone, US $50 billion is spent annually on medical costs related to nonfatal fall injuries, while US $754 million is spent related to fatal falls [17].

Age-related physiological decline significantly increases fall susceptibility [18]. Senescent cells accumulate with age, displaying shortened telomeres and altered signaling that impair tissue repair [19], while sarcopenia progressively weakens skeletal muscles, slowing reflexes and postural control [20]. Changes in visual, vestibular, and proprioceptive pathways compromise precise feedback on body positioning and motion [21]. In parallel, age-related neurodegeneration, including brain atrophy and reduced synaptic connectivity, disrupts the central coordination of motor signals [22]. Endothelial dysfunction and reduced mitochondrial capacity also impair cardiovascular reserves, manifesting as orthostatic hypotension or arrhythmias that lead to sudden drops in cerebral perfusion [23]. Collectively, these age-related physiological and cellular changes not only elevate the risk of falls but also contribute to broader age-related diseases through shared pathophysiological pathways [18].

Given the multisystem involvement of falls, the challenge remains how to optimally screen for fall risk in older adults at the population level. Current best practice is focused on secondary and tertiary fall risk prevention and management following a fall event. The seminal American Geriatrics Society and British Geriatrics Society Clinical Practice Guidelines [24] and recent World Falls Guidelines [15] both recommend annual screening of adults aged 65 years or older for fall risk during routine health care encounters. Currently, there is no universally validated biomarker for predicting falls. While inflammatory mediators (eg, interleukin-6 and tumor necrosis factor-α), hormonal factors (eg, insulin-like growth factor-1 and vitamin D metabolites), and markers of neuromuscular integrity (eg, creatine kinase and neurofilament light chain) have been investigated, there remains no definitive gold standard biomarker for fall risk prediction [25,26]. In the absence of validated biomarkers, fall history in the past 12 months is commonly used as a first-line screener for future fall risk, as research shows that a previous fall is the single best predictor for future falls [27]. The relative risk of falling is 3 times greater for older adults who have previously fallen than for those who have not [28,29], and those who present to health care services with a fall or fall-related injury have a 70% risk of falling again in the next year without intervention [15]. Multifactorial investigations are then recommended for older adults with a positive 12-month fall history to identify specific risk factors, including medication review and assessments for gait, balance, strength, neurological function, cardiovascular status, and visual acuity [24,30].

However, this approach is limited as it does not allow for the identification of at-risk individuals prior to their first fall for targeted primary prevention. Indeed, the sensitivity of common falls-screening algorithms based on the American Geriatrics Society and British Geriatrics Society and World Falls Guidelines, such as the STEADI (Stopping Elderly Accidents, Deaths, and Injuries) algorithm [31], has been shown to be around 60%‐70% with a high false-negative rate in the low-risk categorization group [32,33]. Moreover, current screening approaches rely on patient recall of fall history, which may be impaired by age-related cognitive decline [34], as well as clinician judgment of gait patterns, which can vary depending upon experience, training, and underlying knowledge of gait [35]. Among older adults with a negative 12-month fall history, gait and balance deficits have been identified as the most consistent predictors of future falls [29]. Thus, recognition of gait and balance problems is crucial for identifying persons at risk of first-time falls. However, gait patterns ascertained from clinic and laboratory-based settings often differ from the real-world movement patterns that cause falls, posing an added challenge for clinicians to assess [36]. Furthermore, implementation of physician-led screening in clinical practice has been challenging, with studies finding that over 60% of primary care providers did not routinely ask older adults about fall history or initiate multifactorial falls assessments due to time and resource constraints [31].

In view of these limitations, there has been considerable interest in novel and scalable approaches that can objectively screen and identify individuals at risk of falls, including first-time falls, in real-world settings [36,37]. The ability to predict and prevent falls holds considerable significance in the context of global aging and disease. It is especially critical in Asia, where many regions are experiencing unprecedented population aging. Singapore, in particular, is among the top 5 nations most affected by population aging, with the proportion of older adults in the population expected to reach 40% by 2050 [38]. The standardized incidence rate of fractures among adults aged older than 50 years in Singapore is 314.2 per 100,000, ranking third highest globally and the highest in Asia [39]. By 2050, more than 50% of hip fractures are predicted to occur in Asia [40]. The estimated total economic burden for osteoporotic fractures alone is expected to cost at least SG $289.9 million (SGD $1=US $0.79 as of September 11, 2026) by 2035 [41]. Thus, there is an urgent need for solutions to reduce the health and associated socioeconomic burden of falls and fractures in Singapore and other Asian populations.

While a number of cohort studies have investigated fall or fracture risk factors in Singapore and Asia, the focus of these studies was on general aging [42-44], broad population health [45,46], cancer [46], and eye disease [47], with falls or fractures as a secondary outcome. To bridge this knowledge gap, we implemented a national prospective cohort study of older adults in Singapore to study fall and fracture risk, titled the “Targeted Assessment and Recruitment of Geriatrics for Effective fall prevention Treatments (TARGET)” study. The specific focus of the TARGET study is risk prediction and stratification to support effective therapeutic measures. Its objectives are to (1) develop and validate personalized screening algorithms for assessing the risk of falls (including first-time falls) and fractures among community-dwelling older adults, (2) create risk stratification methods to segment the older adult population into different fall and fracture risk levels with individualized intervention recommendations, (3) conduct cost-effectiveness analyses to support clinical decision-making by identifying the optimal therapeutic window of falls and fractures risk where older adults derive the greatest benefit from interventions, and (4) determine the psychosocial, cognitive, and sociodemographic correlates of the risk of falls and fractures. In this paper, we provide a detailed description of the rationale, design, methodology, and baseline characteristics of the TARGET cohort.

## Methods

### Study Design

TARGET is an ongoing national prospective cohort study of community-dwelling older adults in Singapore, evaluating 3 novel health technology approaches to identify objective functional, cognitive, and imaging-based biomarkers of fall and fracture risk for population-based screening. Between October 2022 and October 2024, a total of 2291 older adults aged 60 years and older were recruited. After study enrollment, participants underwent a comprehensive baseline fall risk assessment (T_0_), followed by 8 follow-up assessments (T_1_-T_8_) via telephone every 3 months for 2 years to prospectively monitor their fall, fracture, and cognitive status. A 2-year follow-up period was selected to inform the development of clinically meaningful predictive models, which can identify individuals at short- (<1 year) and medium-term (1‐2 years) risk for falls for targeted preventive intervention. This duration also provides a balance between collecting sufficient fall events for statistical validity and minimizing participant attrition and recall bias. Ongoing follow-up of the TARGET cohort beyond the initial 2 years is planned to ascertain longer-term fall and fracture risk and outcomes.

### Ethical Considerations

Ethics approval for the study was granted by the National University of Singapore Institutional Review Board (NUS-IRB-2021‐168) and ETH Zurich Ethics Commission (EK 2021-N-145). Written informed consent was obtained from all participants at study enrollment. Participant information sheets and informed consent forms were translated into Chinese, Malay, and Tamil languages by a certified translation company. All participants received an SG $50 (SGD $1=US $0.79 as of September 11, 2026) grocery voucher as a token of appreciation upon successful completion of the baseline home-based assessment. In addition, participants received an SG $140 (SGD $1=US $0.79 as of September 11, 2026) grocery voucher, including the reimbursement for transportation costs, for completion of the baseline clinic assessment.

### Sampling and Recruitment

#### Overview

A multistage random sample of 7501 households with at least 1 older adult resident (Singapore citizen or permanent resident) was purchased from the Department of Statistics of Singapore representing approximately 2.2% of all households with an older adult resident in Singapore in 2022. Household addresses were sampled across all 5 geographic regions of Singapore (Central, East, North, Northeast, and West). To increase the participant count and precision of statistical estimates within minority subgroups, we oversampled addresses with predominant (1) Malay and (2) Indian residents, and with (3) at least 1 resident aged 75 years or older by a factor of 2.

#### Eligibility Criteria

Individuals residing in the random sample of households were eligible for inclusion in the TARGET cohort if they were (1) a Singapore citizen or permanent resident, (2) aged 60‐105 years, (3) spoke at least 1 of the 4 official languages in Singapore (English, Mandarin [or a Chinese dialect], Malay, and Tamil), (4) ambulatory with or without mobility aids, (5) able to see with or without glasses, and (6) able to hear with or without hearing aids. Individuals with at least one of the following criteria at the time of recruitment were excluded: (1) possible delirium or dementia defined as an Abbreviated Mental Test score <6 [48]; (2) blood pressure outside the normal range (defined as ≥200/110 mm Hg or ≥180/100 mm Hg and heart rate >120 per minute or ≤90/60 mm Hg); (3) any symptoms of chest discomfort, breathlessness, dizziness, or profuse sweating; or (4) any acute cardiac event in the past 30 days.

#### Recruitment

An invitation letter was mailed to each household in the random sample stating that participation in the study was completely voluntary, and the study team could be contacted to opt out of the study. Trained field interviewers then conducted home visits 2‐4 weeks later to households that did not opt out. All willing potential participants within each household were first screened to determine their eligibility against the inclusion and exclusion criteria. Multiple residents from the same household were able to participate if they independently met the eligibility criteria. Once participants consented to the study, they were able to complete the baseline assessment on the same day, if convenient, or reschedule the assessment for a later date. Interviewers made up to 5 visits at differing times of day to each residence to recruit participants. Households, which remained uncontactable after 5 visits, were excluded due to nonresponse.

**Figure 1.**
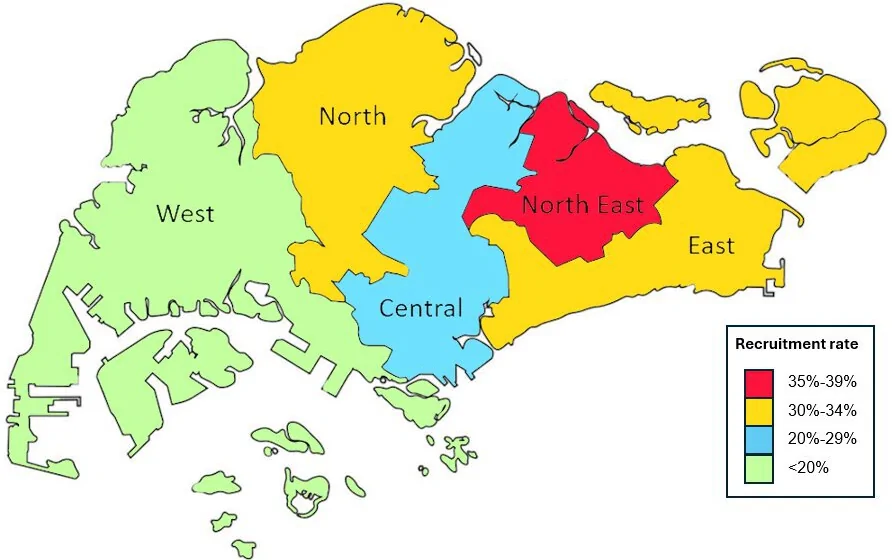
TARGET (Targeted Assessment and Recruitment of Geriatrics for Effective Fall Prevention Treatments) study household recruitment rate by geographic region in Singapore.

At least 1 participant was recruited from 1674 households, giving a household recruitment rate of 22.3% (1674/7501). The household participant yield was 1.3 participants per household (range: 1-4). Anecdotally, the field interviewers reported that the households approached were generally reluctant to interact with strangers, coinciding with active national scam prevention campaigns targeting older adults in Singapore during the recruitment period. Demographic characteristics were compared between household responders and nonresponders. There was no significant difference in the age group of the household reference person (primary resident) between household responders and nonresponders. However, differences in housing type and region were identified. The proportion of private households (ie, residential properties that are not subsidized, built, and managed by the Singapore government) was significantly higher among nonresponders. Response rates also significantly differed by geographic region (Figure 1). The highest response was within the Northeast Region (577/1516, 38.1%) while the West Region (215/1549, 13.9%) yielded the lowest response rate.

### Data Collection

#### Data Collection Overview

Table 1 summarizes key measures, variables, and outcomes. Both primary and secondary data sources were used. Primary data collected included epidemiological survey data (eg, sociodemographic, anthropometric, cognitive, psychosocial, physical, and environmental data), biomechanical data, clinical imaging, and spatial performance data to provide a holistic understanding of fall and fracture risk factors. Additionally, secondary data in the form of electronic medical records (EMRs) containing data on health status, health care use, prescriptions, procedures, and mortality status are being linked from the Singapore Ministry of Health’s TRUST (Trusted Research and Real-World Data Utilisation and Sharing Tech) platform [49] for participants who have consented to this linkage.

**Table 1.** Data collected at baseline and follow-up assessments for the TARGET^a^ study.

Category	Key measures or variables	Baseline (T_0_)	Follow-up (T_1_-T_8_)
1. Fall risk survey			
1.1. Fall history	Number of falls, number of injurious falls	✓	✓
1.2. Anthropometry	Height, weight, blood pressure, and hand grip strength	✓	—^b^
1.3. Sociodemographics	Age, gender, ethnicity, education, employment, housing, and family	✓	—
1.4. Cognitive impairment	Montreal Cognitive Assessment [50]	✓	—
	Telephone Montreal Cognitive Assessment [51]	—	T_2_, T_4_, T_6_, T_8_
1.5. Social network	Modified Lubben Social Network-6 [52]	✓	—
1.6. Quality of life	EQ-5D-5L [53]	✓	—
1.7. Environmental	Modified Life Space Questionnaire [54]	✓	—
1.7. Environmental	Home safety	✓	—
1.8. Functional independence	Modified Barthel Index [55]	✓	—
1.8. Functional independence	Lawton Instrumental Activities of Daily Living [56]	✓	—
1.9. Health	Medical history	✓	—
1.9. Health	Medications	✓	—
1.9. Health	Health and lifestyle behaviors	✓	—
1.9. Health	International Physical Activity Questionnaire-Short Form [57]	✓	—
1.10. Fall risk	Three Key Questions	✓	—
1.10. Fall risk	Fall Risk Questionnaire [58]	✓	—
1.10. Fall risk	Iconographical Falls Efficacy Scale [59,60]	✓	—
1.11. Fracture risk	FRAX tool [61] items including age, gender, weight, height, previous fragility fracture, parental history of hip fracture, smoking, glucocorticoid treatment, rheumatoid arthritis, other secondary causes of osteoporosis, and alcohol	✓	—
1.12. Psychological	Brief Resilience Scale [62]	✓	—
1.12. Psychological	Patient Health Questionnaire [63]	✓	—
2. Gait assessment using wearable sensors	Walking speed, cadence, gait asymmetry, stride time, stride length, stance time, swing time, double-limb support time, heel strike angle, toe-off angle, head stability, postural control, and acceleration patterns	✓	—
3. DXA^c^ scan	Bone mineral density of lumbar spine and proximal hip, whole body 3D composition (bone mineral content, fat mass, lean mass, total mass) of trunk, limbs, and head)	✓	—
4. Spatial navigation assessment	Path integration, egocentric pointing, mapping, associative memory, perspective taking, and Santa Barbara Sense-of-Direction Scale [64]	✓	—
5. Electronic medical record linkage	Outpatient (GP^d^ and polyclinic) and inpatient medical records (diagnosis, procedures, medications, and pathology)	✓	✓

a TARGET: Targeted Assessment and Recruitment of Geriatrics for Effective Fall Prevention Treatments.

b Not available.

c DXA: dual-energy X-ray absorptiometry.

d GP: general practitioner.

#### Baseline Interview

A face-to-face computer-assisted personal interview was conducted with each participant at their residence or other preferred place of convenience using a structured questionnaire to capture sociodemographic, environmental, physical, sensory, medical, cognitive, and psychosocial characteristics related to fall and fracture risk at baseline. Anthropometric measurements (height, weight, and bilateral hand grip strength) were also taken. Responses were collected using the survey software Qualtrics (Qualtrics). The survey was designed by TARGET study investigators and administered by trained field interviewers in the participant’s preferred language (English, Mandarin or Chinese dialect, Malay, or Tamil). The baseline interview took approximately 2 hours to complete, including the screener, consent-taking, and gait assessment detailed in the following text.

#### Gait Assessment

**Figure 2.**
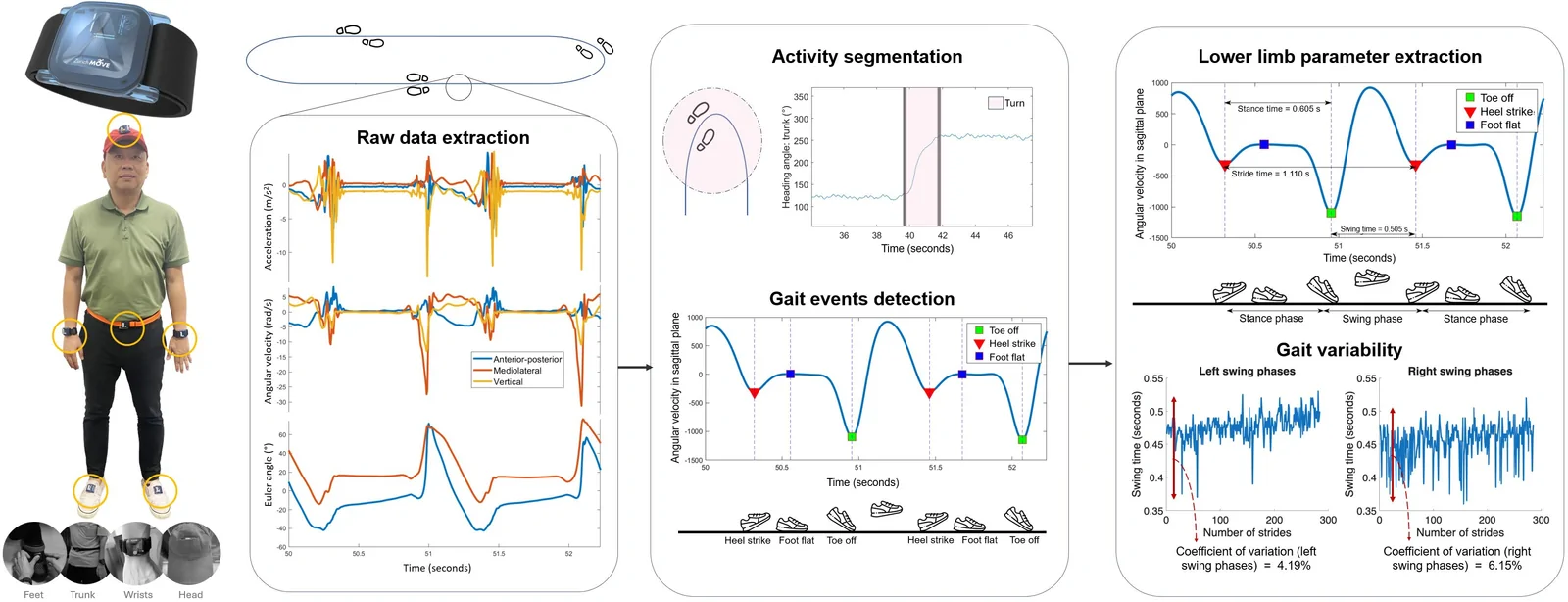
Participants were requested to wear 6 inertial measurement unit sensors (2 each on the feet and wrists, as well as 1 on the trunk and head) while performing a 5-minute walk test under natural settings. The data from the sensors are processed to provide information on turning, gait events, and variability and asymmetry of foot placement while walking.

As a component of the home-based interview, all participants performed a 5-minute walk inside or outside their home, in an open physical space, with the freedom to walk at their preferred pace and navigate turns, while wearing 6 ZurichMOVE inertial measurement units (IMUs) motion sensors [65] affixed to designated anatomical locations (feet, wrists, trunk, and head; Figure 2).

There has been growing research and clinical interest in the use of wearable sensors for identifying fall risk through biomechanical data, specifically, dynamic gait signatures [37,66,67]. Gait and balance assessments are a core component of fall risk assessments in clinical practice. However, existing gold standard assessments such as the Timed Up and Go test and Berg Balance Scale have shown poor predictive accuracy (pooled sensitivity ranging from 0.25 to 0.32) in predicting prospective falls [68,69]. As the majority of falls occur while walking, dynamic gait characteristics such as step length, step time, variability, and asymmetry need to be factored into fall risk assessments for accurate risk prediction [66,70]. Wearable sensor systems can objectively measure a wide range of dynamic gait characteristics, which can then be used to train predictive models for fall risk assessment [71]. IMU sensors, which acquire data using accelerometers and gyroscopes, have gained research and clinical interest as a possible alternative to the current gold standard optical motion capture systems for gait analysis [72]. Compared with optical motion capture, IMU sensors are compact, inexpensive, easier to operate, and can be combined with functional walking tests for gait assessment in the home environment [73].

The IMUs embedded within ZurichMOVE are time-synchronized and have a dynamic range of ±16 g for the accelerometer and 2000° per second for the gyroscope at a sampling frequency of 200 Hz and have been shown to reliably assess gait and identify gait differences in healthy and neurological populations across laboratory settings and real-world environments [74,75]. Triaxial acceleration and angular velocity signals recorded during the walk test are first filtered and analyzed with the validated gait event detection algorithm [70,71], identifying key phases in the gait cycles over the 5 minutes. From these phases, key spatiotemporal parameters such as stride time, length, cadence, and double-limb support time were computed for every participant. Furthermore, intercycle variations are evaluated during the entire walking trial to capture fluctuations and variability in movement patterns, indices associated with deficits in gait, balance, and risk of falling [37,76,77].

#### Fracture Risk Assessment

After the home-based interview, participants who reported that they did not have bilateral hip replacement were invited to a clinic visit at the Singapore Eye Research Institute for a dual-energy X-ray absorptiometry (DXA) scan. Each participant underwent 3 DXA scans (hip, lumbar spine, and whole-body) within a 20‐ to 30-minute session using the Hologic Horizon W scanner (Hologic, Inc). The hip and lumbar spine scans were used to assess areal bone mineral density (aBMD), which was then used to calculate the T-score to evaluate osteoporotic status. Participants with a T-score of ≤−2.5 were classified as osteoporotic, those with a T-score between −2.5 and −1 were classified as osteopenic, and those with a T-score of >−1 were classified as normal, while the whole-body DXA scan captured clinical anthropometric data and body composition, including measurements of fat mass, lean mass, and total mass across the trunk, limbs, and head. These data were used to categorize participants into the low muscle mass group, defined as appendicular lean mass/height^2^ of <7 kg/m^2^ for males and <5.4 kg/m^2^ for females [78]. Additionally, a subset of participants completed 3D-optical body shape scans using the TC2-19M scanner (TC2 Labs LLC), wearing tight-fitting clothing (Spandex top and pants). Participants held an A-line stance for approximately 9 seconds during scanning, which generated a triangular mesh representing their body shape. Data from both the whole-body DXA and 3D optical scans were used to estimate trochanteric soft tissue thickness (TSTT) [79]. TSTT refers to the thickness of the soft tissues, including skin, fat, and muscle, over the greater trochanter of the femur. Clinical risk factors were also gathered, and the risk for major osteoporotic fractures (MOFs) and hip fractures was assessed using the Fracture Risk Assessment Tool (FRAX) without aBMD for the entire cohort. For those who had a DXA scan, FRAX was calculated with the aBMD from the femoral neck. A high risk for MOF was assigned if FRAX-MOF ≥9%, and a high risk for hip fractures was assigned if the FRAX–Hip Fracture (FRAX-HF) score is ≥2% [80]. Age- and sex-adjusted analyses were conducted for all variables, except FRAX-related variables, as age and sex are already incorporated into the FRAX calculation algorithm.

Despite widespread use, studies have shown that aBMD measurements from DXA scans and the 10-year fracture probabilities of hip or MOFs computed using FRAX [61] often fail to detect individuals at risk, as only between 28% and 61% of hip fractures occur in individuals with osteoporosis [62,81,82]. Although FRAX incorporates 12 risk factors and provides country- and ethnicity-specific scores, research suggests that FRAX may be less accurate in Asian populations, compared with Caucasians [63,64]. Finite element models (FEMs) have demonstrated strong potential for improving fracture risk prediction, especially given the limited sensitivity of current standard-of-care methods. Subject-specific FEMs based on computed tomographic scans provide direct estimates of femoral strength and have outperformed aBMD in predicting future fractures, with 1 recent study demonstrating this superiority over a follow-up period of up to 16 years [83-85]. Emerging research from Caucasian cohorts also suggests that FEM-based strength estimates derived from DXA outperform aBMD alone in identifying individuals with hip fractures [86,87]. This body of evidence paves the way for FEM-based fracture risk assessment, particularly when adapted for use with clinical DXA scans for primary screening and with tomographic scans for opportunistic screening, as a more accurate alternative to current methods.

#### Virtual Reality Spatial Navigation Assessment

Participants who completed the DXA scan were also invited to play a virtual reality (VR) spatial navigation game during the clinic visit to assess their cognitive ability. The SPACE (Spatial Performance Assessment for Cognitive Evaluation) is a novel iPad serious game designed to identify deficits in spatial navigation indicative of cognitive impairment and potential fall risk [88]. SPACE has been validated with participants across community and clinical cohorts in Singapore. Studies revealed that SPACE is well suited for older adults [88], and its performance metrics correlate with scores from the Montreal Cognitive Assessment (MoCA); predict hippocampal atrophy beyond age, education, and standard neuropsychological tests [89]; and distinguish between dementia severity levels with high accuracy [90].

Studies have highlighted the potential application of VR technology for identifying markers of cognitive impairment and fall risk [88]. While analysis of cerebrospinal fluid and positron emission tomography can reliably detect dementia-related pathophysiological changes, these methods are invasive and costly, which limits their use for population-based screening [91]. Blood biomarkers are a promising alternative, but these are still undergoing clinical validation and are not yet widely available for routine diagnostic use [92]. In contrast, neuropsychological tests, such as the Mini Mental State Examination and MoCA, are relatively easier to deploy but have shown limited sensitivity to detect early cognitive impairment [93,94]. Critically, most cognitive assessments do not include tests that assess deficits in spatial navigation ability. This lacuna is surprising since spatial disorientation is one of the earliest signs of declining cognitive ability. Indeed, the hippocampus and the entorhinal cortex, which play an instrumental role in navigation, are also among the earliest brain regions to be affected by Alzheimer disease pathophysiology [95-98]. VR technology now offers an accurate, cost-effective, and safe method to assess navigation deficits indicative of cognitive impairment and fall risk in a quick and portable manner [88,99].

In SPACE, participants assume the role of an astronaut tasked with exploring an unfamiliar planet by completing a series of spatial navigation tasks with the help of a companion robot. Each task is specifically designed to probe different aspects of their spatial navigation abilities. SPACE includes a detailed training phase in which players can familiarize themselves with the controls while being assessed on basic visuospatial and motor skills. After the initial training phase, participants perform 5 spatial tasks (ie, path integration, egocentric pointing, mapping, associative memory, and perspective taking) designed to recruit critical brain regions involved in spatial navigation. During gameplay, both performance data and metadata (eg, position and orientation, time, coordinates, and screen interaction) are stored and used to produce individual scores for the various tasks [88].

#### Prospective Follow-Up

Prospective follow-up of the TARGET cohort is currently ongoing. The cohort is being followed up via telephone every 3 months from the baseline interview for 2 years to determine (1) whether they have experienced a fall or fall-related injury over the past quarter, and (2) their cognitive status every 6 months measured using an abbreviated version of the MoCA designed for telephone administration (T-MoCA [51]). Interviewers may also visit participants to conduct the follow-up assessments in person if the participant has difficulties communicating clearly over the phone. As of May 24, 2026, T_6_ (18-month follow-up) is complete for all participants, with 80.5% (1844/2291) successfully followed up (48/2291, 2.1% deaths, and 399/2291, 17.4% lost to follow-up), and 55.3% (1267/2291) of participants have completed T_8_ (24-month follow-up). The study protocol allows for participants with missing follow-up data to be recontacted at subsequent follow-ups to maximize data completeness and cohort retention. Prospective outcomes will be validated against national EMR data. These data will provide granular diagnostic, procedural, and laboratory data on falls, fractures, and broader age-related diseases for the cohort.

#### EMR Linkage

Finally, EMRs for each TARGET cohort member who provided consent for record linkage at baseline (1243/2291, 54.3%) are being obtained through Singapore Ministry of Health’s TRUST platform [49]. TRUST is a secure data exchange platform that enables the fusion and sharing of anonymized health-related research and real-world data for research purposes between public institutions and the public and private sectors. Linked datasets to be requested for the TARGET cohort will include health records (from acute hospitals, polyclinics, general practice clinics, and medical centers), health (emergency department presentations), and birth and death registry (mortality). EMR data will be used to analyze longitudinal pathology, morbidity, and mortality patterns associated with falls and broader age-related diseases in older adults. Analysis will be performed on the deidentified fused datasets in the secure TRUST Workspace.

Table S1 in Multimedia Appendix 1 compares key characteristics between participants who completed and did not complete the clinic visit components (DXA and 3D body scans and SPACE game), and participants who consented and did not consent to the EMR linkage to evaluate potential selection bias. Overall, participants who did not complete the clinic visit components were more likely to be older and have greater ADL/IADL difficulty.

#### Quality Assurance and Control

All researchers were required to complete the Collaborative Institutional Training Initiative online program for research ethics and compliance education. Fieldwork interviewers, including university interviewers and interviewers from a survey research company, were required to undergo training and fieldwork shadowing and to demonstrate competency in the relevant procedures and questionnaire administration prior to being approved to perform household recruitment with participants. Study documents, including participant information sheets, informed consent forms, and questionnaires, were translated into Chinese, Malay, and Tamil languages by a certified translation company. The study team validated the translated documents to ensure that the meaning is consistent with the original version. A computer-assisted personal interview questionnaire was designed in Qualtrics using logic and rules to assist fieldwork interviewers to conduct proper inclusion and exclusion criteria eligibility checks. A pilot study (n=18) was undertaken to test recruitment processes and ensure that interviewers were familiar with the recruitment and interviewing procedures. Data collected by all fieldwork interviewers were checked periodically by key study team personnel. Blood pressure machines and weighing scales were checked and certified by the supplier before being issued to the interviewers. All faulty study instruments are either serviced or replaced depending on the availability of spare parts to ensure measurement accuracy.

### Study Outcomes and Planned Analysis

#### Primary Outcomes

The primary outcomes of interest are the incidence of falls and fractures over the 2-year prospective follow-up period. Falls are defined following the World Health Organization’s definition as “an event which results in a person coming to rest inadvertently on the ground, floor, or lower level” [100]. Fracture events will include both self-reported and electronically validated incident fractures identified during follow-up and through linkage with EMR data. MOF will include fractures of the hip, clinical spine, wrist, and humerus, consistent with the FRAX definition. Hip fractures will be analyzed separately due to their substantial morbidity, mortality, and health care burden among older adults.

Prospective outcomes collected through quarterly follow-up interviews will be validated against linked EMR using relevant diagnostic and procedural coding systems, including ICD (International Classification of Diseases) codes, available within the TRUST-linked datasets. Time-to-event analyses will be conducted to evaluate predictors of incident falls and fractures over the follow-up period.

#### Risk Prediction and Stratification

Primary analyses will focus on the development and validation of novel personalized risk prediction models integrating variables from the epidemiological survey, gait assessment, clinical imaging, SPACE, and EMR data to segment the older adult population into different fall and fracture risk levels. Risk prediction models will be built using state-of-the-art machine learning, deep learning, and statistical techniques. The performance of the novel risk prediction models will be evaluated against existing gold standard assessments (eg, STEADI algorithm, Three Key Questions, and FRAX) and validated against the prospective data. Secondary analyses will focus on (1) developing predictive models of fracture risks among older adults based on holistic body composition parameters obtained from DXA and whole-body 3D shape scans, (2) assessing the performance of SPACE to detect cognitive impairment and its predictive usefulness in detecting falls, and (3) assessing the psychological and sociodemographic correlates of fall risk.

#### Economic Analysis

Cost-effectiveness analyses of screening strategies will be evaluated using decision tree models. The analyses will use Markov models to simulate the long-term costs and quality-adjusted life years over the lifespan. Prospective epidemiological data collected from TARGET will be used as input parameters for event rates and diagnostic accuracy. Performance of novel risk prediction tools (ie, sensitivity, specificity, and accuracy) will be evaluated against current methods of evaluating falls and fracture risk to determine the incremental cost-effectiveness ratios of different screening strategies. A budget impact analysis will determine the short- and medium-term fiscal implications of a national implementation of these strategies.

## Results

### Cohort Characteristics

**Figure 3.**
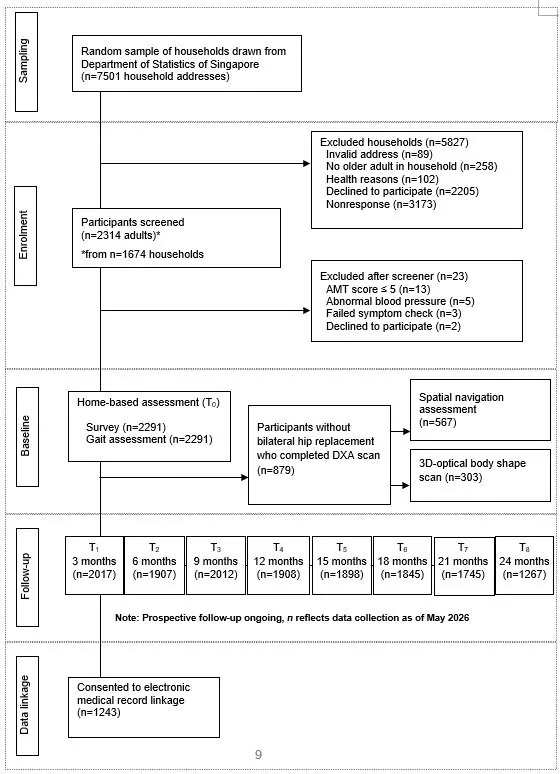
Cohort design and participant flow for the TARGET (Targeted Assessment and Recruitment of Geriatrics for Effective Fall Prevention Treatments) study as of May 2026. AMT: Abbreviated Mental Test; DXA: dual-energy X-ray absorptiometry.

Baseline data collection was completed in October 2024. Baseline cohort characteristics and key preliminary findings to date are summarized in this section. Figure 3 summarizes the TARGET study design and participant flow. Of the 2291 older adults in the TARGET cohort, the majority were female (327/2291, 57.9%) with a mean age of 74.6 (SD 7.9) years at study enrollment (Table 2). Most participants were Chinese (1521/2291, 66.4%), followed by Malay (433/2291, 18.9%), Indian (290/2291, 12.7%), and Other ethnicity (47/2291, 2.1%). Compared with national population reference data from 2023, representing the midpoint of the baseline data collection, the TARGET cohort had a higher proportion of older adults aged ≥75 years, females, and ethnic minorities, and participants from the Northeast region.

**Table 2.** Key sociodemographic characteristics of the TARGET^a^ cohort (N=2291). National data for employment status and education status were not available.

Characteristics	Values, n	Proportion	National proportion (2023)^b^
Age (years)			
60‐64	302	13.2	29.0
65‐69	388	16.9	25.6
70‐74	362	15.8	19.6
75‐79	614	26.8	12.0
80‐84	380	16.6	7.5
85+	245	10.7	6.3
Sex			
Male	964	42.1	47.2
Female	1327	57.9	52.8
Ethnicity			
Chinese	1521	66.4	81.3
Malay	433	18.9	10.5
Indian	290	12.7	6.5
Others	47	2.1	1.6
Geographic region in Singapore			
Central	616	26.9	26.5
East	484	21.1	18.2
North	284	12.4	11.9
Northeast	602	26.3	21.7
West	305	13.3	21.7
Highest education completed			
No formal education	367	16.0	N/A^c^
Primary school	741	32.3	N/A
Secondary school	757	33.0	N/A
Above secondary school	426	18.6	N/A
Employment status			
Full-time	285	12.4	N/A
Part-time	227	9.9	N/A
Not currently working	1582	69.1	N/A
Never worked	197	8.6	N/A
Housing type			
1- or 2-room government-built flat	258	11.3	7.5
3-room government-built flat	570	24.9	18.4
4- or 5-room government-built flat	1355	59.1	55.0
Private housing	108	4.7	19.1
Marital status			
Married	1464	63.9	70.8
Widowed	531	23.2	15.6
Divorced or separated	115	5.1	5.7
Never married	180	7.9	7.9

a Targeted Assessment and Recruitment of Geriatrics for Effective Fall Prevention Treatments.

b From Department of Statistics Singapore 2023 (representing the midpoint of baseline data collection).

c N/A: not applicable.

Over 80% (1898/2291) of the cohort were fully independent at baseline (Table 3). Most participants had mild cognitive impairment (1016/2290, 44.4%; based on MoCA score 18‐25, which represents a level of cognitive impairment greater than expected for age without significant functional impairment) [101], or no cognitive impairment (926/2290, 40.4%; MoCA score ≥26), and 70% (1610/2291) had 2 or more chronic health conditions.

**Table 3.** Summary of select fall and fracture risk factors at baseline of the TARGET^a^ cohort participants (N=2291).

Characteristics	N with data	Values, n	Percentage
Fall history in past 12 months	2291		
At least 1 fall		358	15.6
Recurrent falls (≥2 falls)		104	4.5
Fall-related injury		248	10.8
Hip replacement	2290	48	2.2
Left		22	1.0
Right		22	1.0
Bilateral		4	0.2
Fall risk score (FRQ^b^)	2290		
High risk (≥4)		620	27.1
Low risk (0‐3)		1670	72.9
Cognitive impairment (MoCA^c^)	2290		
None (≥26)		926	40.4
Mild (18-25)		1016	44.4
Moderate (10-17)		340	14.8
Severe (0‐9)		8	0.4
Walking aid used	2288		
Yes		405	17.7
Chronic conditions	2291		
None		302	13.2
≥1		1989	86.8
≥2		1610	70.3
≥3		1096	47.8
≥4		591	25.8
≥5		289	12.6
Functional independence (MBI^d^)	2291		
Independent (100)		1898	82.9
Slight dependence (91-99)		102	4.5
Moderate dependence (61-90)		257	11.2
Severe dependence (21-60)		32	1.4
Total dependence (≤20)		2	0.1
Fear of falling (ICONFES^e^)	2287		
Low concern (10-18)		1462	63.9
High concern (19-40)		825	36.1
Regular prescription medications	2283		
None		441	19.2
1‐4		1276	55.9
≥5 (polypharmacy)		566	24.8

a Targeted Assessment and Recruitment of Geriatrics for Effective Fall Prevention Treatments.

b FRQ: Falls Risk Questionnaire.

c MoCA: Montreal Cognitive Assessment.

d MBI: Modified Barthel Index.

e ICONFES: Iconographical Falls Efficacy Scale.

### Falls Epidemiology and Risk Profile at Baseline

Among the total cohort, 15.6% (358/2291) reported at least 1 fall within the prior 12-month period and 4.5% (104/2291) reported recurrent falls. This is comparable with previously reported fall incidence rates in community-dwelling older adult populations in Singapore [102] but lower than reported rates in the international literature (33%) [15]. Regional differences in falls incidence have previously been identified between Asian and Western countries owing to differences in sociodemographic, geographic, environmental, and cultural factors [103].

### Psychosocial and Sociodemographic Correlates of 12-Month Fall History

Table 4 compares baseline characteristics for TARGET participants by 12-month fall history. Older adults who had fallen in the past 12 months demonstrated significantly poorer physical and psychosocial health across multiple measures at baseline than nonfallers, reinforcing the need for fall risk screening and early intervention in this population. Notably, fallers demonstrated significantly poorer functional independence and quality of life, greater depressive symptoms, higher number of chronic health conditions, and greater fear of falling than nonfallers. However, it is unclear whether preexisting poorer physical and psychosocial health contributed to falls or was a consequence of the fall sequelae. Structural equation modeling analyses are in progress to better understand the causal pathways between falls, functional independence and quality of life, and the potential mediating and moderating mechanisms that can be targeted through interventions. Predictive analyses using the follow-up data are also underway to determine risk factors of prospective falls and fractures in the cohort.

**Table 4.** Comparison of baseline characteristics of TARGET^a^ cohort participants by fall status in past 12 months (N=2291).

Characteristic, mean (SD)	N with data	Faller, n=358	Nonfaller, n=1933	*P* value^b^
Age (years)	2291	75.7 (7.9)	74.3 (7.8)	.02^b^
Anthropometrics				
BMI (kg/m^2^)	2290	24.3 (4.7)	24.6 (4.7)	.26
Blood pressure: systolic	2291	134.5 (18.2)	133.5 (17.2)	.02^b^
Blood pressure: diastolic	2291	75.6 (9.2)	75.8 (9.0)	.47
Heart rate (beats per minute)	2291	75.5 (11.1)	74.8 (10.1)	.27
Left hand grip strength, kg	2245	16.1 (6.0)	18.1 (6.4)	<.001^b^
Right hand grip strength, kg	2259	17.3 (6.3)	19.9 (18.6)	<.001^b^
Functional independence				
IADL^c^	2290	14.0 (3.2)	14.8 (2.5)	.01^b^
ADL^d^	2291	96.1 (9.9)	97.7 (7.5)	.008^b^
Cognitive impairment				
MoCA^e^	2290	22.9 (5.2)	23.3 (4.9)	.01^b^
Physical health				
Chronic conditions	2291	3.2 (2.0)	2.4 (1.7)	<.001^b^
Medications	2283	3.6 (3.1)	2.9 (2.7)	<.001^b^
MET^f^-minutes per week	2272	1374 (1580)	1742 (1717)	.008^b^
Psychosocial				
EQ5D^g^	2290	85.4 (17.8)	91.7 (14.2)	<.001^b^
PHQ-9^h^	2287	2.5 (2.9)	1.4 (2.4)	<.001^b^
Loneliness_Score	2285	3.9 (1.4)	3.6 (1.2)	.002^b^
LSNS-6^i^	2286	13.1 (5.8)	13.9 (5.6)	.001^b^
BRS^j^	2274	22.4 (3.5)	23.0 (2.9)	<.001^b^
Falls				
ICONFES^k^	2287	19.9 (7.2)	16.5 (6.8)	<.001^b^
FRQ^l^	2290	5.4 (2.8)	1.9 (2.3)	<.001^b^

a Targeted Assessment and Recruitment of Geriatrics for Effective Fall Prevention Treatments.

b Significance tests evaluated using multilevel mixed-effects regression models adjusted for clustering at household level, *P*<.05.

c IADL: Instrumental Activities of Daily Living score ranging from 0 to 16 (fully independent).

d ADL: Activities of Daily Living score ranging from 0 to 100 (fully independent).

e MoCA: Montreal Cognitive Assessment score ranging from 0 to 30 (normal cognitive function).

f MET: metabolic estimates.

g EQ5D: EuroQol Index ranging from 1 to 100 (best health).

h PHQ-9: Patient Health Questionnaire–9 score ranging from 0 to 27 (severe depression).

i LSNS-6: Lubben Social Network Score ranging from 0 to 30 (strong social network).

j BRS: Brief Resilience Scale score ranging from 6 to 30 (high resilience).

k ICONFES: Iconographical Falls Efficacy Scale score ranging from 10 to 40 (greater fear of falling).

l FRQ: Fall Risk Questionnaire score ranging from 0 to 12 (high fall risk).

### Gait Assessment

During the home-based interview, 86.4% (1980/2291) of TARGET participants independently completed the full 5-minute walking test, while the remaining 13.6% (311/2291) were able to walk between 3 and 5 minutes. Table 5 compares selected extracted baseline gait metrics between participants with a 12-month history of falls and those without. All participants were analyzed together without adjusting for walking duration as our prior research has shown that 3 minutes of walking covers more than 50 gait cycles (strides) and is sufficient for reliably measuring gait variability [104]. There were no significant differences in the mean and variability of temporal parameters (cadence, stride time, stance time, and double-limb support time) or the spatial measures (step length and gait speed) except for average step length. Among the signal-derived metrics, fallers showed significantly greater variability in the magnitude of foot acceleration (*P*=.02) and significantly lower angular velocity root-mean-square on the trunk sensor (*P*=.02). These preliminary findings suggest that higher gait variability and reduced movement intensity are likely to be associated with a history of falls. These gait parameters are being used for fall risk prediction modeling using machine learning and deep learning models, validated by prospective follow-up of fall status.

**Table 5.** Age- and sex-adjusted comparisons of gait parameters extracted from inertial measurement units sensors by fall status in past 12 months (N=2149).

Gait parameters, mean (SD), range	Faller (n=339)	Nonfaller (n=1810)	*P* value
Temporal parameters			
Mean			
Cadence (steps per minute)	85.7 (18.6), 24.4‐139.5	85.6 (17.8), 23.1‐145.2	.97
Stride time, seconds	1.5 (0.5), 0.9‐4.9	1.5 (0.5), 0.8‐5.2	.75
Stance time, seconds	1.0 (0.5), 0.5‐4.2	1.0 (0.4), 0.5‐4.5	.70
Double limb support time, seconds	0.2 (0.2), 0.1‐1.7	0.2 (0.2), 0.0‐1.9	.64
Variability (coefficient of variation)			
Stride time, %	10.6 (6.1), 2.3‐49.1	9.9 (5.7), 2.1‐55.0	.27
Stance time, %	14.8 (8.4), 3.8‐58.9	13.9 (8.2), 3.0‐72.5	.37
Step time, %	14.2 (8.4), 3.9‐71.4	13.2 (7.8), 2.7‐60.2	.14
Double limb support time, %	37.0 (19.5), 10.0‐143.9	35.2 (20.3), 9.5‐211.9	.35
Spatial parameters			
Mean			
Step length, meters	0.3 (0.2), 0.1‐0.6	0.3 (0.2), 0.0‐0.9	.01^a^
Gait speed, meter per second	0.5 (0.2), 0.0‐1.3	0.5 (0.2), 0.0‐1.3	.30
Variability (coefficient of variation: %RSD^b^)			
Step length	25.4 (10.4), 6.5‐62.9	25.7 (12.2), 4.2‐175.1	.64
Gait speed	22.9 (8.8), 6.6‐55.7	22.2 (9.5), 4.1‐110.2	.43
Signal parameters			
Variability (coefficient of variation: %RSD)			
SD of acceleration magnitude, left foot	29.5 (8.3), 9.9‐62.7	28.3 (7.7) [9.7‐83.8]	.02^a^
Root-mean-square of angular velocity magnitude, trunk	36.2 (12.9), 9.4‐135.0	38.1 (29.0), 9.7‐1121.0	.01^a^

a Values indicate significant differences (*P*<.05).

b RSD: root SD.

### BMD, Body Composition, Morphometry, and FRAX Assessment

Among the 38.4% (879/2291) participants (n=6 were removed for scan artifacts) who underwent DXA scans, 54.9% (483/879) were female. Data from 4 participants were excluded due to lumbar spine scans with motion artifacts. Table 6 summarizes the baseline prevalence of osteoporosis, osteopenia, low muscle mass, and high fracture risk by DXA and FRAX, stratified by gender and ethnicity. A higher percentage of females had osteoporosis than males. However, fewer females were classified as osteopenic than males, and fewer females had low muscle mass than males. Chinese participants had lowest TSTT across both genders compared with Indians and Malays [79]. A higher percentage of females exceeded the FRAX-MOF threshold with aBMD and FRAX-HF threshold with aBMD than males. Similarly, a greater proportion of females surpassed the threshold for FRAX-MOF without aBMD and FRAX-HF without aBMD than males. When comparing ethnicities, the prevalence of osteoporosis, osteopenia, and low muscle mass was the highest in Chinese participants, regardless of gender, compared with their Indian and Malay counterparts [79].

**Table 6.** Prevalence of osteoporosis, osteopenia, low muscle mass, and high FRAX^a^ with aBMD^b^ score (n=879) and FRAX without aBMD score (n=2285), stratified by gender and ethnicity.

Characteristic	Ethnicity			
	Pooled^c^	Chinese	Indian	Malay
Male				
With DXA^d^ scan, n	396	275	49	67
Osteoporotic, %	10.6	11.3	8.2	10.4
Osteopenic, %	51.5	55.6	30.6	50.7
Low muscle mass, %	50.3	53.8	49.0	35.8
High FRAX-MOF^e^, %^f^	21.9	28.4	6.12	5.9
High FRAX-HF^g^, %^f^	67.2	73.8	59.2	44.8
FRAX-related data, n	963	640	118	195
High FRAX-MOF, %^h^	23.9	32.2	10.2	4.6
High FRAX-HF, %^h^	72.6	79.7	65.3	53.3
Female				
With DXA scan, n	483	337	66	78
Osteoporotic, %	13.0	16.0	4.5	7.7
Osteopenic, %	48.9	51.9	36.4	46.2
Low muscle mass, %	39.8	45.7	28.8	24.4
High FRAX-MOF, %^f^	61.7	71.2	50.0	30.8
High FRAX-HF, %^f^	70.4	78.0	59.1	47.4
FRAX-related data, n	1322	880	180	257
High FRAX-MOF, %^h^	71.4	81.1	57.8	47.8
High FRAX-HF, %^h^	79.2	86.4	70.6	60.7

a FRAX: Fracture risk assessment tool.

b aBMD: areal bone mineral density.

c Pooled includes other ethnicities.

d DXA: dual-energy X-ray absorptiometry.

e MOF: major osteoporotic fractures.

f Computed with areal bone mineral density.

g HF: hip fractures.

h Computed without areal bone mineral density.

Table 7 compares baseline aBMD, body composition, morphometry, and FRAX scores by fall status in the past 12 months. TSTT was calculated for the subcohort of 260 participants who had 3D-optical scans (39 fallers and 221 nonfallers, excluding 43 scans with motion artifacts); no significant difference in TSTT was found between fallers and nonfallers. Following age and sex adjustment, the relationships between fallers and nonfallers remained nonsignificant. The mean FRAX scores for both fallers and nonfallers were significantly above the respective clinical thresholds of 9% for FRAX-MOF and 2% for FRAX-HF. Specifically, fallers had significantly higher FRAX-MOF with aBMD scores (mean 13.16, SD 8.34%) than nonfallers (mean 9.97, SD 6.57%), and higher FRAX-HF with aBMD scores (mean 6.11, SD 5.21% vs mean 4.44, SD 4.43%; *P*<.001 for both). Similar patterns were observed without aBMD, with fallers showing higher FRAX-MOF without aBMD scores (mean 14.27, SD 8.58%) and FRAX-HF without aBMD scores (mean 7.17, SD 6.12%) than nonfallers (mean 10.81, SD 6.72% and mean 5.12, SD 4.61%, respectively; *P*<.001).

**Table 7.** Age- and sex-adjusted comparisons of DXA^a^ scanner– and 3D-optical scanner–derived variables, FRAX^b^ with aBMD^c^ scores (n=879), and FRAX without aBMD (n=2285), by fall status in the past 12 months.

Variable	Fallers		Nonfallers		*P* value
Values, n	Mean (SD)	Values, n	Mean (SD)
aBMD (total hip), g/cm^2^	145	0.8 (0.2)	734	0.8 (0.2)	.76
aBMD (total hip), g/cm^2^^d^	145	0.8 (0.1)	734	0.8 (0.1)	.21
aBMD (femoral neck), g/cm^2^	145	7 (0.2)	734	0.7 (0.1)	.47
aBMD (femoral neck), g/cm^2^^d^	145	7 (0.1)	734	0.7 (0.1)	.44
aBMD (lumbar spine), g/cm^2^	145	0.9 (0.2)	730	1.0 (0.2)	.79
aBMD (lumbar spine), g/cm^2^^d^	145	1.0 (0.2)	730	1.0 (0.2)	.33
T-score (total hip)	145	-1.2 (1.2)	734	−1.3 (1.1)	.52
T-score (total hip)^d^	145	-1.2 (1.1)	734	−1.3 (1.1)	.28
Appendicular fat mass, kg	145	10.5 (3.5)	734	10.3 (3.8)	.37
Appendicular fat mass, kg^d^	145	10.3 (3.4)	734	10.4 (3.4)	.81
Appendicular lean mass, kg	145	15.6 (3.8)	734	16.2 (3.9)	.07
Appendicular lean mass, kg^d^	145	16.3 (2.4)	734	16.1 (2.4)	.39
Whole-body total fat mass, kg	145	23.5 (7.1)	734	23.3 (7.1)	.95
Whole-body total fat mass, kg^d^	145	23.2 (6.8)	734	23.4 (6.8)	.82
Whole-body total lean mass, kg	145	37.1 (7.6)	734	38.4 (7.8)	.06
Whole-body total lean mass, kg^d^	145	38.5 (4.9)	734	38.1 (4.9)	.47
Muscle mass, kg/m^2^	145	6.1 (1.0)	734	6.3 (1.1)	.09
Muscle mass, kg/m^2^^d^	145	6.3 (0.9)	734	6.2 (0.9)	.87
TSTT^e^, cm	39	3.4 (1.1)	221	3.5 (1.3)	.85
TSTT, cm^d^	39	3.9 (1.1)	221	3.5 (1.1)	.55
FRAX^f^-MOF^g^, %^h^	145	13.2 (8.3)	734	10.0 (6.6)	<*.001*^k^
FRAX-HF^i^, %^h^	145	6.1 (5.2)	734	4.4 (4.4)	<*.001*^k^
FRAX-MOF, %^j^	357	14.3 (8.58)	1928	10.8 (6.7)	<***.****001*^k^
FRAX-HF, %^j^	357	7.2 (6.1)	1928	5.1 (4.6)	<***.****001*^k^

a DXA: dual energy X-ray absorptiometry.

b FRAX: fracture risk assessment tool.

c aBMD: areal bone mineral density.

d Adjusted for age and sex.

e TSTT: trochanteric soft tissue thickness.

f FRAX: fracture risk assessment tool.

g MOF: major osteoporotic fractures.

h Computed with bone mineral density.

i HF: hip fractures.

j Computed without areal bone mineral density; the Mann-Whitney *U* test was used to compare the distributions of key variables between fallers and nonfallers.

k Values in italics indicate significant differences (*P*<.05).

With the body shapes from the 3D-optical scans used to develop a soft tissue database for Asian adults across genders and ethnicities, DXA-derived FEMs will be enhanced by incorporating this soft tissue information to more accurately predict fracture risk in a patient-specific manner. These models will be prospectively validated against fracture outcomes obtained from electronic health records. Through this validation, we aim to determine optimal thresholds for identifying high-risk individuals. Establishing these thresholds will, in turn, inform screening protocols, ensuring that they are cost-effective and scalable for implementation within Singapore’s health care system.

### SPACE

Of 2291 participants, the subset of 567 (24.7%) participants who completed SPACE had a higher average MoCA score (mean 25.9, SD 3.6), scoring 2.8 points above the remainder of the cohort. All tasks in SPACE significantly correlated with MoCA scores. Robust comparisons using the M-estimator median method revealed no significant differences between fallers and nonfallers for all tasks in SPACE; however, a large group imbalance was noted (Table 8). Planned analyses using the TARGET cohort will focus on evaluating the combined predictive usefulness of SPACE and gait metrics in detecting falls and fractures. SPACE has been shown to predict hippocampal atrophy beyond age, education, and neuropsychological assessment [89], and can accurately distinguish between dementia severity levels (area under the curve=0.94‐0.95) [90].

**Table 8.** Age- and sex-adjusted comparisons of SPACE^a^ performance by fall status in the past 12 months (N=567).

SPACE tasks, median (n)	Faller	Nonfaller	*P* value
Training time	271.2 (98)	269.7 (468)	.97
Path integration	207.5 (80)	205.7 (393)	.82
Pointing	79.9 (66)	77.3 (340)	.84
Mapping	0.7 (66)	0.5 (339)	**.** *03* ^b^
Memory	100.0 (66)	100.0 (339)	.98
Perspective taking	45.7 (84)	49.4 (447)	.42

a SPACE: Spatial Performance Assessment for Cognitive Evaluation.

b Value in italics indicates significant differences (*P*<.05).

## Discussion

### Principal Findings

To the best of our knowledge, TARGET is the largest prospective cohort study in Asia to jointly investigate fall and fracture risk factors among a national population of community-dwelling older adults. Overall, the TARGET cohort represents a relatively healthy community-dwelling older adult population, with over 80% retaining full functional independence at baseline. However, there is some evidence of early or moderate age-related decline: 70% (1610/2291) of the cohort reported at least 2 chronic conditions, 59% (1356/2290) exhibited mild-moderate cognitive impairment, and among those who underwent DXA scans, 50% (440/879) had osteopenia. Falls were reported by 15% (358/2291) of participants in the past year, whereas 85% (1933/2291) remained fall-free. Thus, TARGET is an ideal cohort for investigating the risk factors that predispose relatively healthy older adults to transition to first-time falls and fractures. Through EMR linkage, this can be extended to study the onset of broader age-related diseases.

By leveraging novel health technology data, TARGET aims to identify novel patterns in gait, cognition, and skeletal fragility as key markers of fall and fracture risk, and potentially other age-related diseases with shared pathophysiology. Data from the wearable IMU sensors capture subtle deficits in balance, stride variability, and reaction times, which cannot be detected through clinical observation alone, providing ecologically valid measurements of real-world performance where traditional clinical thresholds of 0.8 meters per second [15] or 1.0 meter per second [78] may no longer be applicable. These data can be particularly informative for age-related diseases where gait and balance impairments play an early or significant role such as Parkinson disease, stroke, multiple sclerosis, and peripheral neuropathy [105]. SPACE tasks probe spatial orientation that is often overlooked by traditional cognitive tests, with important implications for the early detection of falls as well as dementia [88,89]. The DXA-derived FEM-based models may also identify more subtle skeletal vulnerabilities that might be missed by standard T-score classifications [83,86,87], offering potential insights into other skeletal conditions such as degenerative joint disease or metabolic bone disorders.

These novel health technology data are strengthened by clinical and laboratory data from EMR linkage, which add essential pathological and biochemical context, allowing for correlations between physiological changes and real-world health outcomes. Finally, robust epidemiological survey data capturing sociodemographic, environmental, psychosocial, and functional parameters enable the identification of synergistic risk factors such as social isolation and psychological resilience that may affect fall and fracture risk and accelerate age-related decline. Collectively, these rich and novel cohort data place TARGET in a strong position to identify emerging biomarkers and systemic interactions that may predict falls and fracture risk and potential other broader age-related pathophysiology.

Critically, insights generated by the TARGET study are likely to make a significant contribution to the development of personalized community-based screening and intervention frameworks for Asian populations, where differences in genetics, lifestyle, health care systems, and environmental factors have been shown to influence both fall and fracture risk profiles and outcomes; yet, where there remains a critical gap in region-specific evidence and scalable screening solutions. While the fracture risk component of TARGET relies on specialized equipment such as DXA scans and 3D body scanning, the fall risk component uses portable, low-cost technologies (eg, IMU sensors) that allow trained nonclinical personnel to conduct on-site assessments. This expanded approach has the potential to reduce substantial burden and costs within the health care sector, with whom the responsibility of health screening traditionally lies, by broadening access in community settings.

### Limitations

The TARGET cohort is not a fully representative national sample of community-dwelling older adults in Singapore. Specifically, TARGET contains a higher proportion of individuals aged 75‐84 years and of Malay and Indian backgrounds, reflecting the intentional oversampling of these minority groups to ensure statistical power for subgroup analyses. Furthermore, it excludes certain older adult subgroups, such as those not ambulatory or with possible delirium or dementia. While this approach allows for more nuanced investigation of fall and fracture risk factors within these subpopulations, it may limit the generalizability of the findings to the broader older adult population in Singapore. Additionally, not all participants contributed data for every health technology assessed in the study, particularly for the DXA scan and VR assessment. As a result, some analyses will be based on subcohorts, which could affect the statistical power and introduce potential bias in technology-specific findings. Finally, it is acknowledged that our household response rate of 22.3% (1674/7501) is lower than expected compared with previous national surveys of older adults we have conducted in Singapore (35% response rate) [106]. This lower response rate may introduce selection bias, as individuals who chose to participate could differ systematically from those who declined, potentially in terms of health status, sociodemographic characteristics, or other relevant factors.

## Supplementary material

10.2196/89285 Multimedia Appendix 1 Comparison of baseline characteristics of TARGET (Targeted Assessment and Recruitment of Geriatrics for Effective Fall Prevention Treatments) cohort participants by completion of DXA, 3D body scan, and SPACE (Spatial Performance Assessment for Cognitive Evaluation) assessments.

## Abbreviations

aBMD: areal bone mineral density
DXA: dual-energy X-ray absorptiometry
EMR: electronic medical record
FEM: finite element model
FRAX: Fracture Risk Assessment Tool
FRAX-HF: Fracture Risk Assessment Tool–Hip Fracture
FRAX-MOF: Fracture Risk Assessment Tool–Major Osteoporotic Fracture
*ICD*: *International Classification of Diseases*
IMU: inertial measurement unit
MoCA: Montreal Cognitive Assessment
MOF: major osteoporotic fracture
SPACE: Spatial Performance Assessment for Cognitive Evaluation
STEADI: Stopping Elderly Accidents, Deaths, and Injuries
T-MoCA: MoCA designed for telephone administration
TARGET: Targeted Assessment and Recruitment of Geriatrics for Effective Fall Prevention Treatments
TRUST: Trusted Research and Real-World Data Utilisation and Sharing Tech
TSTT: trochanteric soft tissue thickness
VR: virtual reality
