# Supplementary material for: Rationale, Design, and Baseline Characteristics of the TARGET (Targeted Assessment and Recruitment of Geriatrics for Effective Fall Prevention Treatments) Cohort of Older Adults for Assessing Fall and Fracture Risk: Prospective Cohort Study
Source: JMIR Aging. 2026 Sep 25;9:e89285. doi: 10.2196/89285 (PMC13614221; doi:10.2196/89285)
Supplement: Supplementary file 1 [file aging-v9-e89285-s001.docx]

**Multimedia Appendix 1**

**Table S1a. Comparison of Baseline Characteristics of TARGET Cohort Participants by Completion of DXA, 3D Body Scan and SPACE Assessments.**

| Characteristics | Completed DXA (n=879) | Did not complete (n=1412) | *p*-value^*^ | Completed 3D Body Scan (n=260) | Did not complete (n=2031) | *p*-value^*^ | Completed SPACE (n=566) | Did not complete (n=1,741) | *p*-value^*^ |
| --- | --- | --- | --- | --- | --- | --- | --- | --- | --- |
| Age, mean (SD) | 72.3 (7.5) | 76.0 (7.8) | **<.000*** | 71.6 (7.2) | 75.8 (7.9) | **<.000*** | 71.5 (7.1) | 75.6 (7.8) | **<.000*** |
| Gender, n (%) |  |  | **.006*** |  |  | .275 |  |  | **.014*** |
| Male | 395 (44.9%) | 569 (40.3%) |  | 116 (44.6%) | 848 (41.8%) |  | 258 (45.6%) | 706 (40.9%) |  |
| Female | 484 (55.1%) | 843 (59.7%) |  | 144 (55.4%) | 1,183 (58.2%) |  | 308 (54.4%) | 1,019 (59.1%) |  |
| Ethnicity, n (%) |  |  | .100 |  |  | **<.000*** |  |  | **.032*** |
| Chinese | 615 (70.0%) | 906 (64.2%) |  | 93 (35.8%) | 1,428 (70.3%) |  | 411 (72.6%) | 1,110 (64.3%) |  |
| Malay | 136 (15.5%) | 297 (21.0%) |  | 82 (31.5%) | 351 (17.3%) |  | 80 (13.1%) | 353 (20.5%) |  |
| Indian | 113 (12.8%) | 177 (12.5%) |  | 80 (30.8%) | 210 (10.3%) |  | 62 (11.0%) | 228 (13.2%) |  |
| Others | 15 (1.7%) | 32 (2.3%) |  | 5 (1.9%) | 42 (2.1%) |  | 13 (2.3%) | 34 (2.0%) |  |
| IADL, mean (SD) | 0.9 (2.0) | 2.2 (3.4) | **<.000*** | 0.9 (2.0) | 2.8 (3.1) | **<.000*** | 0.6 (1.4) | 2.1 (3.3) | **<.000*** |
| ADL, mean (SD) | 98.2 (6.2) | 96.9 (8.9) | **.001*** | 98.4 (4.5) | 97.3 (8.4) | **.008*** | 98.6 (5.8) | 97.0 (8.6) | **.001*** |
| Cognitive impairment (MoCA), mean (SD) | 25.1 (4.2) | 22.2 (5.0) | .189 | 24.5 (4.4) | 23.1 (5.0) | .352 | 26.9 (3.6) | 22.4 (5.0) | .253 |
| Medications, mean (SD) | 2.8 (2.7) | 3.2 (2.8) | .410 | 3.2 (3.0) | 3.0 (2.7) | **.001*** | 2.6 (2.7) | 3.2 (2.8) | .407 |
| MET-minutes/week, mean (SD) | 1801.0 (1751.0) | 1600.9 (1656.9)) | **.011*** | 1856.6 (2035.7) | 1654.7 (1646.7) | .094 | 1871.3 (1730.7) | 1614.1 (1680.2) | **.003*** |
| ICONFES, mean (SD) | 15.9 (6.4) | 17.6 (7.2) | .729 | 15.8 (6.1) | 17.1 (7.0) | **.003*** | 15.4 (6.1) | 17.5 (7.1) | **<.000*** |
| FRQ, mean (SD) | 1.9 (2.3) | 2.8 (2.9) | **<.000*** | 2.2 (2.4) | 2.5 (2.7) | .069 | 1.8 (2.2) | 2.7 (2.8) | **<.000*** |

**Table S1b. Comparison of Baseline Characteristics of TARGET Cohort Participants by Consent for EMR Linkage.**

| Characteristics | Consent to EMR data linkage  (n=1,243) | Did not consent to EMR data linkage  (n=1,048) | *p*-value^*^ |
| --- | --- | --- | --- |
| Age, mean (SD) | 74.3 (7.9) | 74.8 (7.8) | .130 |
| Gender, n (%) |  |  |  |
| Male | 560 (45.1%) | 404 (38.6%) | **<.000*** |
| Female | 683 (54.9%) | 644 (61.4%) |  |
| Ethnicity, n (%) |  |  | **.002*** |
| Chinese | 786 (63.2%) | 735 (70.1%) |  |
| Malay | 242 (19.5%) | 191 (18.2%) |  |
| Indian | 192 (15.5%) | 98 (9.4%) |  |
| Others | 23 (1.8%) | 24 (2.3%) |  |
| IADL, mean (SD) | 1.7 (2.9) | 1.7 (3.2) | .098 |
| ADL, mean (SD) | 97.2 (8.3) | 97.7 (7.7) | .164 |
| Cognitive impairment (MoCA), mean (SD) | 23.4 (4.8) | 23.1 (5.1) | .435 |
| Medications, mean (SD) | 3.1 (2.8) | 3.0 (2.7) | **.047*** |
| MET-minutes/week, mean (SD) | 1816.1 (1782.2) | 1513.5 (1572.9) | **<.000*** |
| ICONFES, mean (SD) | 16.9 (7.0) | 17.1 (6.9) | .244 |
| FRQ, mean (SD) | 2.5 (2.8) | 2.4 (2.6) | .056 |

^*^ Significance tests evaluated using logistic regression with cluster-robust standard errors to account for within-household clustering, *P*<0.05
